# Rehabilitation Procedures in the Management of Parkinson's Disease

**DOI:** 10.1155/2015/824056

**Published:** 2015-12-21

**Authors:** Alessandro Picelli, Talia Herman, Serene S. Paul, Laurie A. King

**Affiliations:** ^1^Neuromotor and Cognitive Rehabilitation Research Center, Department of Neurological, Biomedical and Movement Sciences, University of Verona, 37134 Verona, Italy; ^2^Center for the Study of Movement, Cognition and Mobility, Department of Neurology, Tel Aviv Sourasky Medical Center, 64239 Tel Aviv, Israel; ^3^Department of Physical Therapy, University of Utah, Salt Lake City, UT 84108, USA; ^4^The George Institute for Global Health, Sydney Medical School, The University of Sydney, Sydney, NSW 2050, Australia; ^5^Department of Neurology, Oregon Health & Science University, Portland, OR 97239, USA

Parkinson's disease (PD) is a chronic and progressive neurodegenerative condition characterized by a progressive depletion of dopaminergic neurons in the substantia nigra pars compacta [1]. It affects approximately 7 million, primarily elderly, people worldwide [2]. Motor cardinal signs of PD include bradykinesia, rigidity, resting tremor, and postural instability, as well as deterioration of muscle strength, cardiorespiratory fitness, performance of balance, gait, and mobility tasks [2–4]. In addition to motor symptoms, people with PD may suffer from nonmotor complications such as sensory complaints, autonomic dysfunction, fatigue, apathy, sleep disturbances, depression, and cognitive decline (i.e., executive function) [2, 4]. Disability can occur at all stages of PD leading to decreased independence, inactivity, social isolation, and reduced quality of life by performance of activities of daily living and various aspects of mobility such as gait, transfers, balance, and posture [5].

The management of PD has traditionally centered on drug therapy with levodopa viewed as the “gold standard” treatment [5]. However, even with optimal medical management, people with PD experience deterioration in bodily functions as well as limitations in daily activities and participation [5, 6]. On these bases, the role of rehabilitation has gained a prominent place in the overall management of PD. Specifically, there is a move towards using rehabilitation procedures as an adjunct to pharmacological and surgical treatments with an emphasis on multidisciplinary management of this multidimensional condition [5, 7]. Rehabilitation for PD aims to maximize functional ability and minimize secondary complications by focusing on improving balance, posture, gait, upper limb function, physical capacity, and cognition, as well as minimizing falls, in order to optimize individuals' independence, safety, and well-being, thereby enhancing quality of life [7].

In this special issue of Parkinson's disease, we invited investigators to submit their contributions about rehabilitation procedures in the management of people with PD. We particularly focused on articles proposing some evidence for innovative rehabilitation protocols (including treatments based on motor-cognitive approaches) and comparing the effects of different rehabilitation therapies. Other articles explored rehabilitation strategies such as physical activity, physiotherapy, electromechanical and robot-assisted training, virtual reality, and telerehabilitation. Finally, we focused on the role of cognitive dysfunction in PD rehabilitation. This issue highlights the emerging and important role that rehabilitation plays in the management of motor and nonmotor symptoms of PD. The wide range of topics included in this issue demonstrates the complexity of the disease and the need for a multidisciplinary approach to rehabilitation for PD.

*Alessandro Picelli* *Alessandro Picelli* *Talia Herman* *Talia Herman* *Serene S. Paul* *Serene S. Paul* *Laurie A. King* *Laurie A. King*

## References

[1] Fahn S., Sulzer D. (2004). Neurodegeneration and neuroprotection in Parkinson disease. *NeuroRx*.

[2] Uhrbrand A., Stenager E., Pedersen M. S., Dalgas U. (2015). Parkinson's disease and intensive exercise therapy—a systematic review and meta-analysis of randomized controlled trials. *Journal of the Neurological Sciences*.

[3] Speelman A. D., Van De Warrenburg B. P., Van Nimwegen M., Petzinger G. M., Munneke M., Bloem B. R. (2011). How might physical activity benefit patients with Parkinson disease?. *Nature Reviews Neurology*.

[4] Müller B., Assmus J., Herlofson K., Larsen J. P., Tysnes O.-B. (2013). Importance of motor vs. non-motor symptoms for health-related quality of life in early Parkinson's disease. *Parkinsonism & Related Disorders*.

[5] Tomlinson C. L., Herd C. P., Clarke C. E. (2014). Physiotherapy for Parkinson's disease: a comparison of techniques. *The Cochrane Database of Systematic Reviews*.

[6] Nijkrake M. J., Keus S. H. J., Kalf J. G. (2007). Allied health care interventions and complementary therapies in Parkinson's disease. *Parkinsonism & Related Disorders*.

[7] Tomlinson C. L., Patel S., Meek C. (2013). Physiotherapy versus placebo or no intervention in Parkinson's disease. *The Cochrane Database of Systematic Reviews*.

